# El Nino Southern Oscillation (ENSO) impact on tuna fisheries in Indian Ocean

**DOI:** 10.1186/2193-1801-3-591

**Published:** 2014-10-09

**Authors:** Palanisamy Satheesh Kumar, Gopalakrishna N Pillai, Ushadevi Manjusha

**Affiliations:** Central Marine Fisheries Research Institute, Kochi, Kerala 682 018 India

**Keywords:** Climate, Indian Ocean, ENSO, El Nino, La Nina, Sea surface temperature, Tuna

## Abstract

El Nino Southern Oscillation (ENSO) is an important driver of interannual variations in climate and ecosystem productivity in tropical regions. However, detailed information about this important phenomenon of the Indian Ocean is scarce. Consequently, the objective of this study is to improve understanding of the impact of warm event El Nino and cool event La Nina on annual tuna landings from the Indian Ocean from 1980 to 2010. In this study, maximum tuna landings were recorded during a weak El Nino year (1456054 t in 2006) and during a weak La Nina year (1243562 t in 2000), although the lowest tuna catch was followed during the strong El Nino year (1204119 t in 2009) and during a strong La Nina year (706546 t in 1988). Validation of predicted tuna landings and SST were showing a significant positive correlation (*p* < 0.01) was observed all the major tuna species except Southern Bluefin Tuna. Whereas the other relationships such as sea level pressure, Wind actions; Zonal Wind (U), Meridonial Wind (V), and Scalar Wind (W) are less well-defined. In contrast with principal component analysis we find that Principal Components 1 explains 75.5% of the total variance and suggest that sea surface temperature plays a major role in determining tuna availability in the region especially during warm event El Nino years; landings in Indian Ocean tend to be optimum SST 25 to 26°C in ENSO event. Our results confirm the ENSO impact on climate, tuna abundance and production in the Indian Ocean. However, among the oceanic variables SST explained the highest deviance in generalized additive models and therefore considered the best habitat predictor in the Indian Ocean followed by sea level pressure and Winds (U, V, W).

**Electronic supplementary material:**

The online version of this article (doi:10.1186/2193-1801-3-591) contains supplementary material, which is available to authorized users.

## Introduction

Climate change presents an emerging challenge to the sustainable management of tuna fisheries, and robust information on essential to ensure future sustainability (Nicol et al. 2013). Climate change affects the survival, growth, reproduction, recruitment and distribution of individuals within a species, but impacts can also be shown at the level of populations, communities, or entire ecosystems (Brander 2007; Lehodey et al. 1997, 2003; Pillai and Satheeshkumar 2012, 2013). Recent changes in the distribution of a number of fish species can be ascribed with high confidence to regional climate variability, such as the El Nino Southern Oscillation (ENSO) and anomalies (Brander 2007). The ENSO phenomenon is regarded as the primary cause for interannual climatic variability around the globe. The economies of many countries, mainly in tropical regions, depend on the presence and intensity of this phenomenon (Suarez et al. 2004; Lehodey et al. 2006). Therefore, it is important to have the means to allow predictions regarding time, intensity and potential damage (Suarez et al. 2004).

El Niño events are associated with physical and biological changes in our oceans that affect fish abundance and distribution. The result of analysis of fisheries and environmental variables has well established that tuna distribution and abundance are closely associated with physical changes in the ocean. Sea Surface Temperature (SST), SST anomalies, dissolved oxygen concentration, illumination, wind speed, current movements, depth of mixing layer, upwelling and food availability are major factors which are influencing the growth, and convergence zone (Diaz and Markgraf 1992; Lehodey et al. 1997, 2003; Chavez et al. 1999). Scientific evidence supports the conclusion that climate change is already altering marine ecosystems (Lehodey et al. 1997, 2003; Nicol et al. 2013). Primary and secondary production and structure of marine communities are affected by the increase in ocean temperature, increased stratification of the water column and changes in the intensity and timing of coastal upwelling with consequent impacts on fish migration patterns, recruitment, growth, distribution, abundance and predator and prey relationship (Sissener and Bjorndal, 2005; McIlgorm et al. 2010). El Niño events provide an excellent opportunity to observe and evaluate how changes in microhabitats influence the tuna fish distribution. However large gaps in our knowledge remain, particularly the implications of climate change for the large and complex food web that supports tuna populations and their fisheries, and whether climate change may alter the interactions between tuna fishing and ecosystem structure and function in the Indian Ocean. Identifying changes in tuna distribution will increase our understanding of the habitat requirements of important marine resources and will help us better manage our tuna fisheries in the future.

On a large scale, distribution and abundance of tuna are determined by ocean temperature (Mullen 1992). However, detailed information on climate variability and its impact on tuna resources in the Indian Ocean is very scanty. Recent studies have found that tuna population dynamics is influenced by the ENSO phenomenon (Lehodey et al. 2008). Interestingly, few investigations have been conducted on the relationship between fluctuations in oceanic environmental conditions and tuna catch in the Pacific regions and the Panama bight during El Nino years (Lehodey et al. 1997; 2003; Pedraza and Dıaz-Ochoa 2006). Studies have indicated that Pacific Ocean tunas have thermal limits for maximum abundance of Yellowfin between 20° to 30°C and Skipjack between 20° to 29°C (Stretta 1991). The earlier predictions of ENSO (several months beforehand), are a major consequence of discovering the position of the highest abundance of skipjack tuna in the Western Pacific, This result has significant implications for the commercial tuna fishing industry (Lehodey et al. 1997).

Many studies based on tuna fishery statistics have investigated the relationships of the variability of tuna abundances with different oceanic parameters in the major oceans (Pedraza and Dıaz-Ochoa 2006; Chen et al. 2005; Lehodey et al. 2008; Song et al. 2009; Brander 2012). Certainly, understanding how the oceanic environment affects distribution of tuna species is an essential step towards ecosystem-based management of fisheries, which is increasingly becoming a basic requirement in management policy (Pillai and Satheeshkumar 2013). Time series data and its analyses are the prerequisites for investigating long-term fluctuations in fish populations and the relationships between populations and environmental variables. Therefore, the aim of this study is to analyze the effect of El Nino and La Nina events on tuna availability of the Indian Ocean from 1980 to 2010. We examined the relationship between tuna landing and environmental variables such as Sea Level Pressure (SLP) and Sea Surface Temperature (SST), Zonal Wind (U), Meridonial Wind (V), and Scalar Wind (W) in the tropical Indian Ocean at an interannual scale. Multivariate statistical analysis such as Principal Component Analysis (PCA) and Non- Multidimensional Scale Plot (MDS) was implemented to investigate the relationships of these environmental variables with landings of major tuna species.

## Materials and methods

### Tuna fishery data

**Figure 1:**
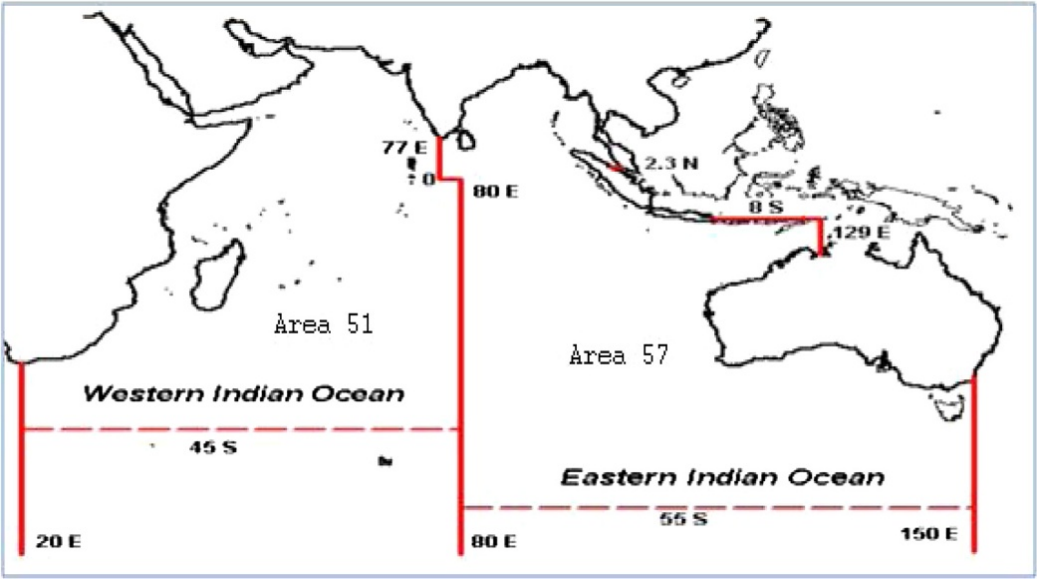
**Indian Ocean fishing area.**

The Indian Ocean is designated conventionally as an area between 25° N and 40° S and between 40° E and 115° E. The database used in this present study is the Indian Ocean Tuna Commission (IOTC) based on nominal catch data of tuna from the Western Indian Ocean fishing area (51) and Eastern Indian Ocean (57) as depicted in Figure 1. We used landing statistics of tuna catch in the Indian Ocean as a proxy for the abundance or availability of the fish in the Indian Ocean waters fishing by the long line, bait boat, gill net, Pole and line, Purse seine and other gear. It would probably be better to have standardized landings by fishing effort. Unfortunately data on fishing effort spent for tuna fishing were not available or not sufficient information available from the (IOTC) or any other agencies, in view of the fact that landings could be a good indicator of tuna abundance.

### Environmental data

Environmental data were collected (Indian Ocean area between 25° N and 40° S and between 40° E and 115° E) from the National Oceanic and Atmospheric Administration (NOAA). Sea surface temperature (SST), Sea level pressure (SLP), Zonal Wind (U), Meridonial Wind (V), and Scalar Wind (W) data from 1980 to 2010 was retrieved from the International Comprehensive Ocean- Atmosphere Data Set (ICOADS) http://icoads.noaa.gov/ carried aboard NOAA-series polar-orbiting satellites. These data are in a resolution of 2° square, for correlating with the Indian Ocean tuna catches, all month-wise oceanographic parameters were averaged into year wised (Plisnier et al. 2000); the analysis of this study was performed to evaluate the long term outcomes from January 1980 to December 2010. Standardized Anomalies of oceanographic parameters were calculated by subtracting the climatological monthly cycle from the data (Plisnier et al. 2000).

**Figure 2:**
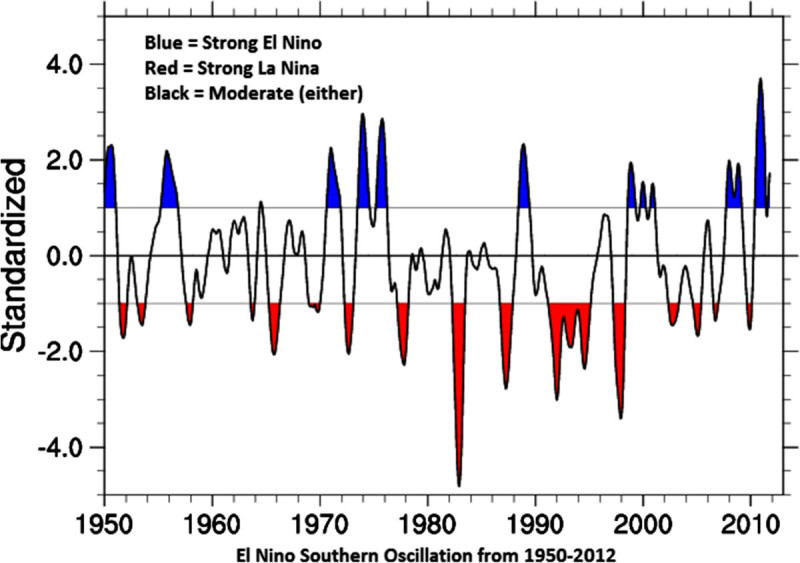
**Recent years El Nino Southern Oscillation index source:**https://climatedataguide.ucar.edu/climate-data/overview-climate-indices.

ENSO phenomenon is regarded as the main cause for inter annual climatic variability around the world (Plisnier et al. 2000; Suarez et al. 2004). ENSO is an irregular low-frequency oscillation between a warm event (El Nino) and cold event (La Nina) state that evolves under the influence of the dynamic interaction between atmosphere and ocean. Events are defined as 5 consecutive months at or above the +0.5° anomaly for warm (El Nino) events and cold (La Nina) events. The threshold is further broken down into weak (with a 0.5 to 0.9 SST anomaly), moderate (1.0 to 1.4) and strong (>1.5) events. The list of El Nino and La Nina Years retrieved from (http://www.ggweather.com/enso/oni.htm) is given in Table 1. The relevant large scale climatic phenomena include irregular long term climatic regime shifts, as well as the quasi periodic oscillation between El Nino and La Nina events in the eastern tropical Pacific as a result of the ENSO (Figure 2). El Nino and La Nina events cause physical changes across wide regions of the Pacific and Indian Oceans that have a good number documented effect the abundance and spatial distribution of several commercially important fish stocks (Miller 2007).

**Table 1:** **The list of El Nino and La Nina Years during 1951-2010**

El Nino			La Nina		
Weak	Mod	Strong	Weak	Mod	Strong
1951	1986	1957	1950	1954	1955
1963	1987	1965	1956	1964	1973
1968	1994	1972	1962	1970	1975
1969	2002	1982	1967	1998	1988
1976		1991	1971	1999	2010
1977		1997	1974	2007	
2004		2009	1984		
2006			1995		
			2000		
			2011		

Source: http://ggweather.com/enso/oni.htm.

### Statistical analysis

Coefficient of correlation (r) was performed to understand the relationship between the various oceanic parameters and to test the significance of the models. Means and standard deviations were computed for each oceanic parameters (SST; Wind U, V, Z and SLP). All these statistical analyses were performed using SPSS statistical (Version 13 for Windows XP, SPSS, and Chicago, IL, USA). Multivariate statistical techniques such as Non-Multidimensional Scale plot (MDS) and Principal Component Analysis (PCA) have widely been used as unbiased methods in analysis of water quality and relationship between marine organisms; water quality (Quadir et al. 2007; Ischen et al. 2008; Satheeshkumar and Khan 2012), phytoplankton characteristics (Wang et al. 2006); benthos characteristics (Satheeshkumar 2012). Based on the groups obtained cluster analysis, species having the greatest contribution to this distribution were determined using similarity percentage program PAST (statistical Version 1.93 for Windows XP).

### Principal component analysis

PCA technique extracts the eigenvalues and eigenvectors of the covariance matrix of original variables. The new axes lie along the directions of maximum variance (Shrestha and Kazama 2007). It reduces the dimensionality of the data set by explaining the correlation amongst a large number of variables in terms of a smaller number of underlying factors, without losing much information (Alberto et al. 2001). The PCA can be expressed as.

$$Z_{\mathrm{ij}}={\mathrm{pc}}_{i1}x_{1j}+\ldots \;\ldots \;\ldots ..{\mathrm{pc}}_{\mathrm{im}}X_{\mathrm{mj}}$$

Where z is the component score, pc is the component loading, x is the measured value of the variable, i is the component number, j is the sample number, and m is the total number of variables.

### Non-multidimensional scale plot

MDS is a set of related statistical techniques often used in information visualization for exploring similarities or dissimilarities in data. Ordination plots produced by MDS analyses were used to classify cases into categorical dependent values. One of its objectives is to determine the significance of different variables, which can allow the separation of two or more naturally occurring groups.

### Data treatment

Most of the multivariate statistical methods require variables to confirm the normal distribution, thus the normality of the distribution of each variable was checked by analyzing kurtosis and skewness statistical tests before multivariate statistical analysis is conducted (Lattin et al. 2003; Satheeshkumar and Khan 2012). The original tuna catch data and environmental data demonstrated values of kurtosis ranging from -1.96 to 10.36 and skewness values ranging -1.91 to 4.21 indicating that the data was not normally distributed. Since most of the values of kurtosis and skewness were >0, the raw data of all variables were transformed in the form x’ = log 10(x). After transformation, the kurtosis and skewness values ranged from -0.612 to 1.37 and –1.06 to 1.22 respectively, indicating that all the data were normally distributed or close to being normally distributed. In the case of PCA, and MDS, all log-transformed variables were also z-scale standardized to minimize the effects of different units and variance of variables and to render the data dimensions (Singh et al. 2004).

## Results and discussion

### Climatic variability in the Indian Ocean

#### Wind

**Figure 3:**
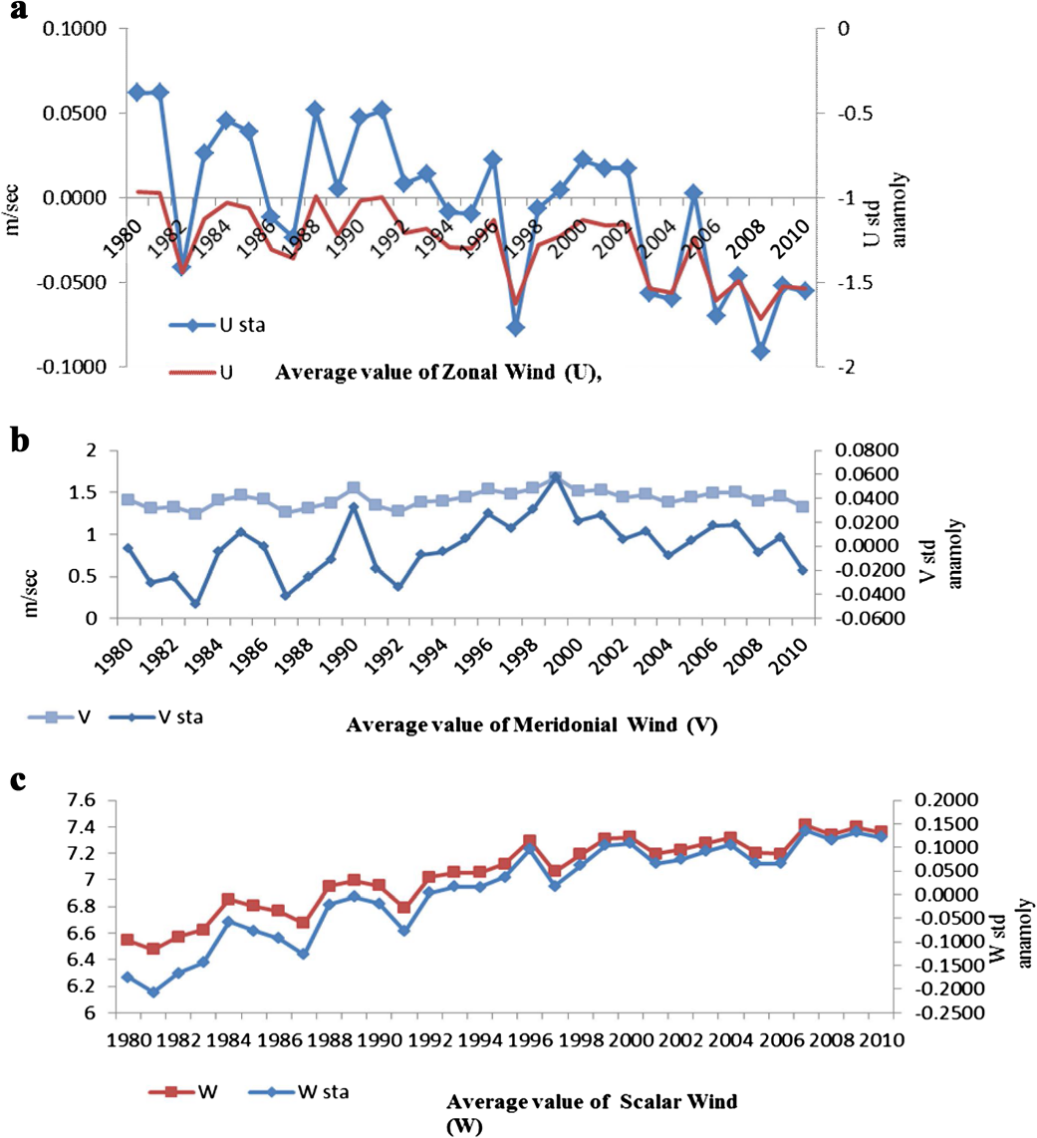
**Indian Ocean wind action and standardized anomalies during 1980-2010. a** Mean value of U Wind and Standardized anomalies. **b** Mean value of V Wind and Standardized anomalies. **c** Mean value of W Wind and Standardized anomalies.

Figure 3 shows the Indian Ocean wind action and standardized anomalies during 1980–2010. Along the Indian Ocean coast wind forcing plays a major role in upwelling phenomena, a significant positive correlation (*p* < 0.01) obtained between meridonial wind and scalar wind (Table 2). A negative correlation was obtained between zonal wind and meridional wind (r = -0.546; *p* < 0.01) and meridional wind and SLP (r = -0.581; *p* < 0.01). The positive anomaly of meridional and scalar wind triggers anomalous downwelling oceanic Rossby waves, thereby deepening the thermocline and resulting in advection of warm waters in the Indian Ocean. Recent observations of ENSO have shown that the Indian Ocean is consistently warm and its warm pool is expanding, particularly in the recent decades (Achuthavarier et al. 2012).

**Table 2:** **Correlation of El Nino years tuna catches with oceanic parameters**

	SLP	U	V	W	SST	YFT	BET	SKJ	ALB	SBF	LOT	FRI	BLT	FRZ	KAW
SLP	1.00														
U	-0.33	1.00													
V	-0.27	-0.41	1.00												
W	-0.52	-0.38	0.63*	1.00											
SST	-0.58	-0.38	0.38	0.75*	1.00										
YFT	-0.51	-0.33	0.54	0.79*	0.68*	1.00									
BET	-0.28	-0.33	0.67	0.72*	0.51	0.90	1.00								
SKJ	-0.61	-0.30	0.63*	0.85**	0.84**	0.87	0.83	1.00							
ALB	-0.22	-0.04	0.29	0.56	0.52	0.16	0.18	0.38	1.00						
SBF	0.79**	-0.04	-0.52	-0.80	-0.68	-0.74	-0.71	-0.79	-0.33	1.00					
LOT	-0.43	-0.39	0.72*	0.92**	0.86**	0.62	0.67	0.83	0.69	-0.74	1.00				
FRI	-0.23	-0.44	0.77**	0.84**	0.71	0.68	0.81	0.80	0.57	-0.72	0.90	1.00			
BLT	-0.22	-0.51	0.89**	0.86**	0.62	0.69	0.85	0.82	0.48	-0.67	0.88	0.93	1.00		
FRZ	-0.43	-0.52	0.69*	0.95**	0.86**	0.73	0.73	0.86	0.57	-0.73	0.97	0.88	0.91	1.00	
KAW	-0.45	-0.45	0.68*	0.95**	0.86**	0.73	0.77	0.89	0.59	-0.77	0.98	0.91	0.93	0.99	1.00

*Correlated at 5% significance level.

**Correlated at 1% significance level.

#### Sea surface temperature

**Figure 4:**
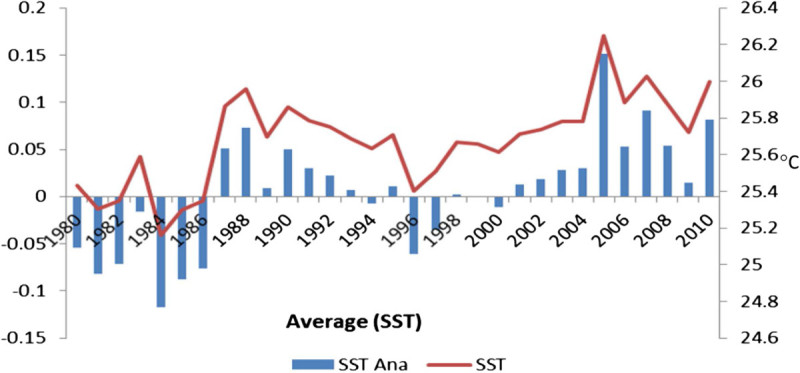
**Indian Ocean SST and standardized anomalies during 1980-2010.**

El Nino phenomena were not active during 1966 to 1976 but thereafter became more active, especially in 1990. Values of SST anomaly were negative in 1970–1980s and late 1996–2000 (Plisnier et al. 2000), although they were positive in the mid 1980s and 2001–2010. Figure 4 depicts the variation of interannual anomaly of the Indian Ocean during El Nino and La Nina years. A significant positive correlation (r = 0.502; *p* < 0.01) obtained between SST and scalar wind indicates that SST is largely influenced by scalar wind in this region (Table 2). A negative correlation was observed between SST with SLP (r = -0.400; *p* < 0.05) and zonal wind (r = -0.356; *p* < 0.05). One key finding of this study is that interdecadal fluctuations contribute strongly to tropical Indian Ocean SST variability in cold events (La Nina) and warm events (El Nino). Based on our observation on Indian Ocean SST variation, El Nino increased average ocean temperature in the region 25.16 to 26.24°C during 1980–2010. During the second half of the twentieth century the Indian Ocean exhibited a rapid rise in sea surface temperature. Figure 4 represented the higher values of SST anomalies were observed during 2005 (0.15) and 1988 (0.07) and lowest value was observed in 1984 (-0.11). The time difference between maxima of SST anomalies in the Indian Ocean is approximately 8–17 years, indicating slow eastward propagation of the multi-decadal climate signal. Climate change is likely to affect regional tuna fisheries in two major ways: by raising average ocean surface temperatures to levels currently experienced during medium-intensity El Niños (Timmermann et al. 1999) and by increasing year-to-year climate variability.

#### Sea level pressure

**Figure 5:**
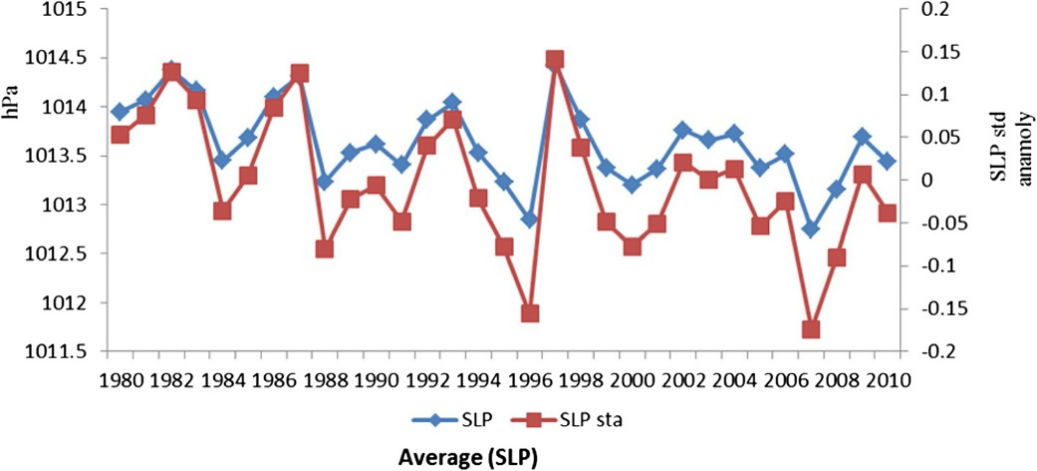
**Sea level pressure and standardized anomalies during 1980-2010.**

Indian Ocean spatial evolution of SLP displayed in Figure 5, are striking during 1980–2010. The negative correlation was obtained between SST and scalar wind (r = -0.382; *p* < 0.05), the maximum values of the SLP anomalies were observed during 1997 (0.140), 1987 (0.127) and later in 2009 (0.070) and lowest values were observed in 2007 (-0.174). The surface conditions must then reflect subsurface thermal conditions throughout the mechanical wind action linked to SLP anomalies patterns and gradients (Tourre et al. 2007). From the slow evolution of the SLP anomalies patterns and gradients in the Indian Ocean display an overall slow meridonial evolution of the eastern Indian Ocean into the western Indian Ocean. Copsey et al. (2006) presented the evidence that the Indian Ocean warming was associated with local increases in sea level pressure (SLP). The negative correlation observed between meridional wind, SST and SLP especially over the La Nina periods support the role of the SLP in modifying the thermodynamic forcing that appears to be dominating the air–sea interaction processes.

### Indian ocean tuna fisheries and ENSO

#### Tuna vs El Nino year and relationship with climate variability

**Figure 6:**
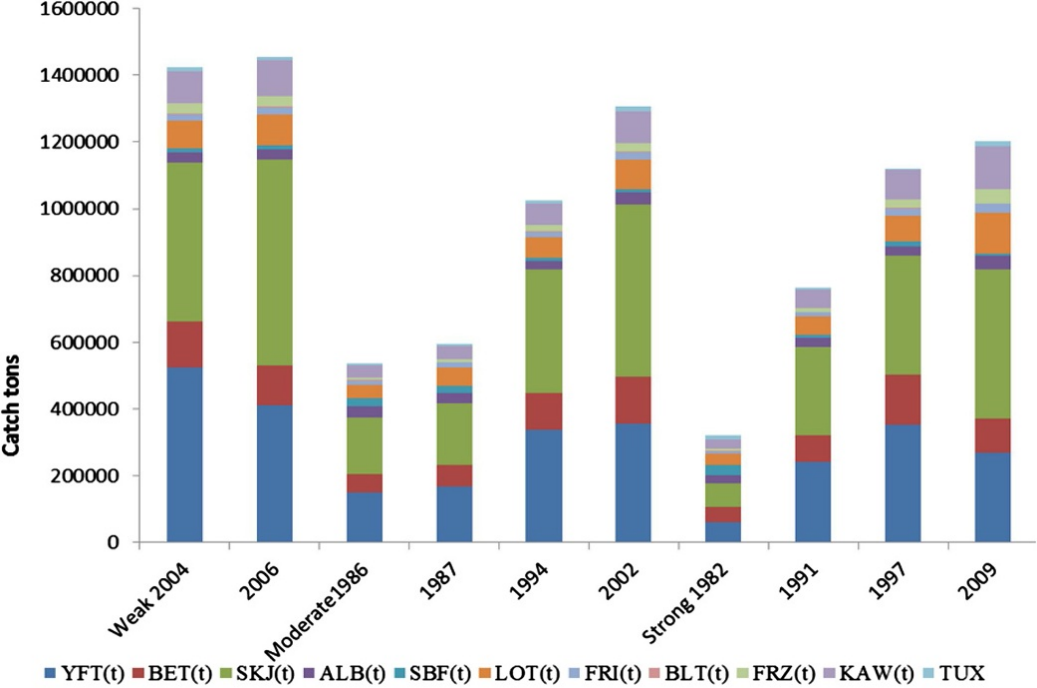
**Tuna landings in Indian Ocean during El Nino years.**

**Figure 7:**
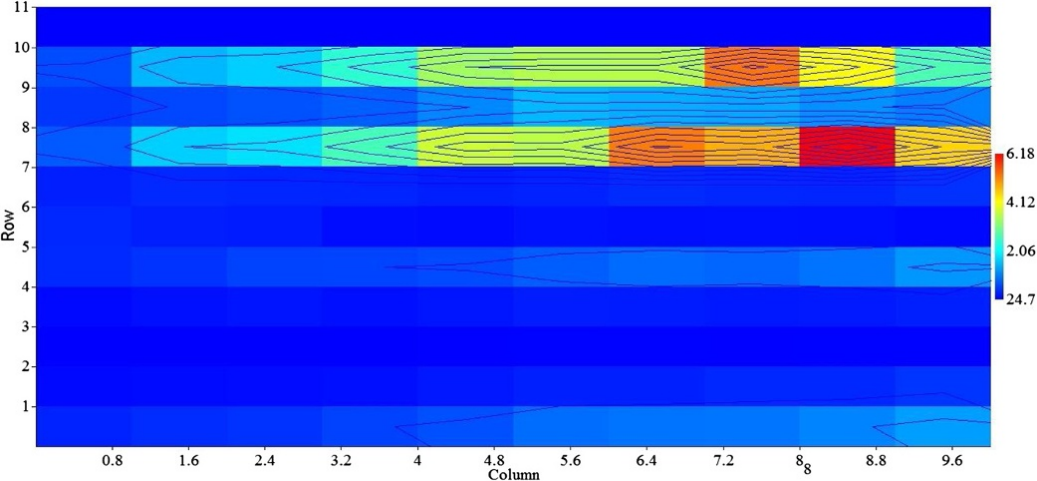
**Two dimensional scale polt analysis of El Nino SST and tuna catch.**

Figure 6 shows tuna fish landings in the Indian Ocean during El Nino years, the highest tuna catch was reported during weak El Nino years in 2006 (14,56054 t) and in 2004 (14,25640 t); the reduced tuna catch was recorded during the moderate El Nino years 1987 (5,95495 t), in 1994 (10,24744 t) and in 2002 it has increased as (13,08704 t). The lowest tuna catch was observed during the strong El Nino year in 1982 (3,21289 t), in 1997 (11, 32282) and in 2009 (12,04119 t) respectively. The recent moderate and strong El Nino years, the maximum tuna catch was recorded compared to 1980 to 1990s, its due to increased number of fishing crafts (increased fishing effort) and human manpower. Until now, correlations have been useful to demonstrate that climate-related variability of fish populations is the rule rather than the exception (Lehodey et al. 2006). The SST indicated a strong significant correlation with skipjack SKJ (r = 0.842; *p* <0.01), long tail tuna LOT (r = 0.855; *p* <0.01), kawa kawa KAW (r = 0.867; *p* <0.01), and yellowfin YFT (r = 0.685; *p* <0.05). While a strong positive significant correlation (r = 0.794; *p* < 0.01) was obtained between SLP and SBT, Meridonial wind (V) had a significant positive correlation with frigate tuna FRI (r = 0.77; *p* <0.01), bullet tuna BLT (r = 0.891; *p* <0.01), FRZ (r = 0.685; *p* <0.01), KAW (r = 0.68; *p* <0.01), SKJ (r = 0.636; *p* <0.05), and LOT (r = 0.721; *p* <0.05). Scalar wind (W) had a highly significant positive relationship with YFT (r = 0.794; *p* <0.01), SKJ (r = 0.855; *p* <0.01), LOT (r = 0.939; *p* <0.01), FRI (r = 0.842; *p* <0.01), BLT (r = 0.867; *p* <0.01), FRZ (r = 0.952; *p* <0.01), and with KAW (r = 0.95; *p* <0.01) respectively (Table 2). The El Nino warm event affects the distribution, abundance, and catchability of Indian Ocean tuna fisheries. Two dimensional scale plot of the data matrix was revealed to identify the impact of warm EL Nino event SST and SST anomalies on tuna landings, both variables used to generate an overview over a large data matrix. Missing values are plotted as blanks. The sparse matrix plot was observed in different colour bar depending on its relative tuna catch during El Nino Year (Figure 7), the diverse colour bars were noted with includes contours. The length of colour bar represents the sparsity pattern of the matrix confirms the influence of SST and SST anomalies on tuna production during the El Nino year. Lan et al. (2012) were reported the effects on the catch per unit effort (CPUE) of yellowfin tuna catch by the Taiwan longline fishery in the Arabian Sea. CPUE showed positive correlations with SST and dipole mode index (DMI) and chl-*a*, especially a long-term positive correlation for the regular longline fishery in 1998–2005 with a periodicity of 2 years. Catch rates of longline fisheries appear to be associated with the depth of tuna habitats; deep thermocline depth caused a high CPUE for the deep longline fishery (Lan et al. 2012).

#### Tuna vs La Nina year and relationship with climate variability

**Figure 8:**
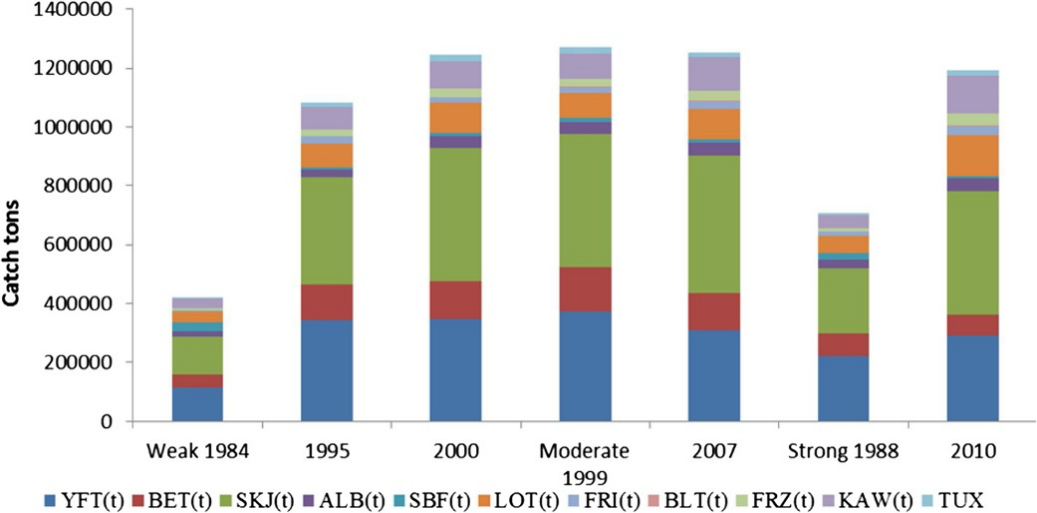
**Tuna landings in Indian Ocean during La Nina years.**

**Figure 9:**
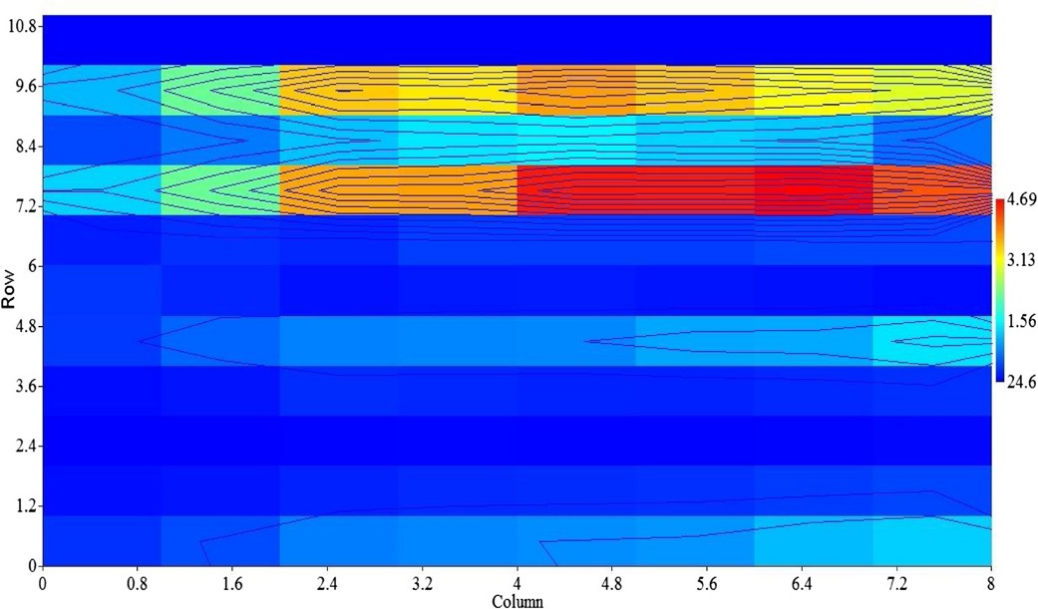
**Two dimensional scale polt analysis of El Nino SST and tuna catch.**

Tuna landings of the Indian Ocean during La Nina Year 1980 to 2010, which are considered to be representative of extreme environmental conditions, are presented in (Figure 8). Tuna catch during the weak La Nina year’s in the Indian Ocean was 10, 82471 t in 1995 and 12,43562 t in 2000 and in moderate La Nina years 12,52186 t in 2007. However, the lowest tuna catch recorded during the strong La Nina year in 1988 (70, 6546 t), and in 2010 (11, 91828 t) respectively. Based on the La Nina years from (1980 to 2010), annual tuna landings in the Indian Ocean were correlated with SST, SLP, Winds (U, V and W). Sea surface temperature (SST) indicating a highly significant relationship with the following tuna species, BLT (r = 0.976; p < 0.01), ALB (r = 0.786; p < 0.05), FRI (r = 0.786; p < 0.05), and had a negative correlation with SBF (r = -0.738; p < 0.05). Zonal wind (U) positively correlated with SBF (r = 0.881; *p* < 0.01), and negatively correlated with FRI (r = -0.952; *p* < 0.01), BLT (r = -0.95; *p* < 0.01), FRZ (r = -0.74; *p* < 0.05), and with KAW (r = -0.738; *p* < 0.05) respectively. Scalar wind (W) had a highly significant relationship with SKJ (r = 0.905; *p* < 0.01), ALB (r = 0.90; *p* < 0.01), FRZ (r = 0.976; *p* < 0.01), KAW (r = 0.98; *p* < 0.01), BLT (r = 0.762; *p* < 0.01), and indicating a negative correlation with SBF (r = -0.738; p < 0.05) respectively (Table 3). Figure 9 shows a two dimensional scale plot of the data matrix was revealed to identify the impact of cold La Nina event SST and SST anomalies on tuna landings. The sparse matrix plot was observed in different colour bar depending on tuna catch, the similar colour bars were noted with scaled down contours. The stretch of colour bar indicates the sparsity pattern of the matrix confirm the influence of SST and SST anomalies on tuna production during the La Nina year.

**Table 3:** **Correlation of La Nina years tuna catches with oceanic parameters**

	SLP	U	V	W	SST	YFT	BET	SKJ	ALB	SBF	LOT	FRI	BLT	FRZ	KAW
SLP	1.00														
U	0.19	1.00													
V	0.09	-0.08	1.00												
W	-0.29	-0.77**	0.43	1.00											
SST	-0.40	-0.86**	-0.19	0.70	1.00										
YFT	-0.09	-0.49	0.78*	0.78*	0.37	1.00									
BET	-0.01	-0.26	0.84**	0.62	0.13	0.89	1.00								
SKJ	-0.28	-0.69	0.54	0.96**	0.59	0.91	0.77	1.00							
ALB	-0.16	-0.68	0.28	0.90**	0.76*	0.62	0.49	0.82	1.00						
SBF	0.24	0.78*	-0.14	-0.84**	-0.75*	-0.83	-0.54	-0.87	-0.71	1.00					
LOT	-0.18	-0.81*	0.01	0.88**	0.80*	0.62	0.29	0.81	0.85	-0.89	1.00				
FRI	-0.06	-0.86**	0.20	0.80*	0.72*	0.79	0.53	0.82	0.66	-0.96	0.85	1.00			
BLT	-0.02	-0.94**	-0.04	0.76*	0.87**	0.47	0.19	0.66	0.77	-0.80	0.91	0.86	1.00		
FRZ	-0.19	-0.87**	0.18	0.94**	0.80*	0.63	0.38	0.86	0.89	-0.85	0.97	0.84	0.92	1.00	
KAW	-0.24	-0.90**	0.19	0.95**	0.84**	0.68	0.43	0.88	0.88	-0.90	0.96	0.88	0.92	0.99	1.00

*Correlated at 5% significance level.

**Correlated at 1% significance level.

Previous studies have shown that the distribution of albacore is affected by SST (Chen et al. 2005). Based on the oceanic parameter model, skipjack tuna habitat selection was significantly (*p* < 0.01) influenced by SST ranging from 20.5 to 26°C in the Pacific Ocean (Mugo et al. 2010). Loukos et al. (2003) suggested significant large scale changes of skipjack habitat in the equatorial Pacific due to increased ocean temperature caused by global warming. Such a Scenario could expand available habitat for warm water pelagic to higher latitudes (Cheung et al. 2009). The distribution of the principal market tuna species, population, and recruitment of the tuna stock is influenced by the ENSO variability and changes in oceanic parameters such as SST, DO and salinity. However, as these species are more widely distributed and have extended spawning grounds in both east and western Indian Ocean, the relationship with ENSO is more complex (Pillai and Satheeshkumar 2013). Our results based on SST indicate that El Nino significantly (*p* < 0.01) influenced by skipjack tuna habitat in the Indian Ocean. Brill et al. (1999) also suggest SST to be the limiting factor of large adult yellowfin tuna near the Hawaiian Islands, whereas SST, salinity, SLP, current movement, and dissolved oxygen concentration influence spawning activities of yellowfin tuna (Romena 2001). Primary productivity in the eastern and western Indian Ocean would decline due to increased stratification between warmer surface waters and cold deep waters. The decreased upwelling would lead to a decline in the bigeye and adult yellowfin tuna population, the species targeted by the longline (Lehodey et al. 1997). Of all the factors considered SST should be considered as the main factor of Albacore, skipjack and yellowfin tuna is most sensitive to fluctuation of oceanic temperature. It is noticed from the present study that the distribution of tuna and tuna like species would respond to displacement of SST, wind movements and convergence zone that might provide a foundation for the prediction of good tuna fishing grounds.

### Multivariate statistical analysis

#### Principal component analysis

Impact of ENSO on tuna fisheries in Indian Ocean was performed by the time series analysis using multivariate analysis. PCA were applied to standardized log-transformed data set (Tuna fish landing value and environmental parameter) to identify the latent factors. The objective of this analysis was primarily to create an entirely new set of factors much smaller in number when compared to the original data set in subsequent analysis. Before applying PCA, correlation analysis was carried out. This was utilized to find an internal structure and assist to identify the influence of environmental parameters on Indian Ocean tuna distribution. The highest positive correlation existed between SST, SLP and Wind (U, V, W) and tuna catch during El Nino and La Nina years, the Zonal wind (U) negatively correlated with all the tuna species except SBF tuna (Tables 2 and 3).

**Figure 10:**
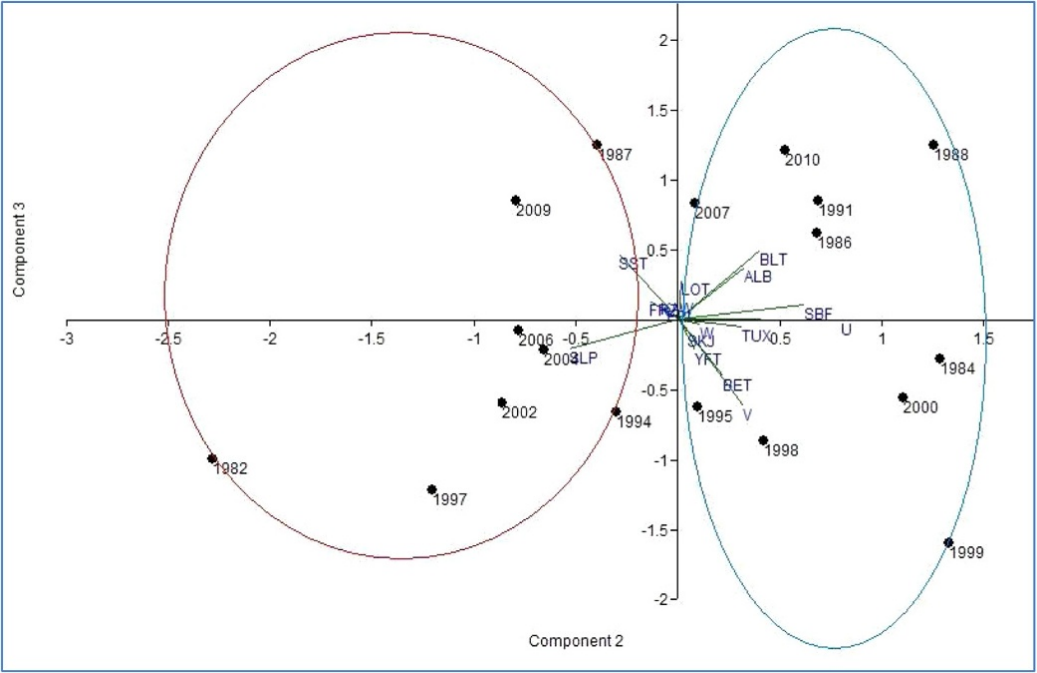
**Principal component analysis derived annual observations of Oceanic variables and tuna landings of Indian Ocean.**

The results of the PCA are given in (Figure 10). Four principal components accounting for 99.56% of the total variation are retained on the basis of the eigenvalue greater- than-one rule. The first two principal components explain 75.5% and 21.16% of the variance, respectively. The third and fourth principal components are considerably less important, explaining only 2.39% and 0.51% of the variance, respectively. Therefore, we consider only the first two components, which account for a large proportion of the variation in the data (97.1% of the variance). The cluster dendrogram displayed two groups according their principal components. The Cluster A showed the relationship between tuna catch during El Nino years (weak, moderate, strong) and environmental parameters. The Figure illustrates that cluster A included 1982, 1994, 1997, 2002, 2004, 2006 characterize negative values in principal components, respectively; 1987, 1991 and 2009 characterize the positive values. Cluster B was characterized by La Nina years (weak, moderate, strong) tuna catch and the relationship between the Indian Ocean oceanographic parameters. These time series data/observations showed 1984, 1995, 1998, 1999 and 1986, 2007, 2010 characterize the positive and negative values in principal components.

However, from the PCA results, it may be presumed that during the strong El Nino year (1982, 1997 and 2009) the tuna landings are decreased in the Indian Ocean due to warm El Nino climatic variability and during the weak El Nino years (2004, 2006), the tuna catches may increase due to SST and SLP optimum value. Climate change is likely to affect regional tuna fisheries in two major ways: by raising average ocean surface temperatures to levels currently experienced during medium-intensity El Niños (Timmermann et al. 1999) and by increasing year-to-year climate variability. In the present study, the highest tuna catch was recorded in the Indian Ocean during 2004 and 2006, these findings are also supported by the tuna catch in a moderate La Nina year (2007) highest tuna catch was recorded in the Indian Ocean and 1191828 t in 2010 (strong La Nina year) was landed. This group (Cluster B) thus displays on opposite pattern to Cluster A.

### Multidimensional scale plot analysis

**Figure 11:**
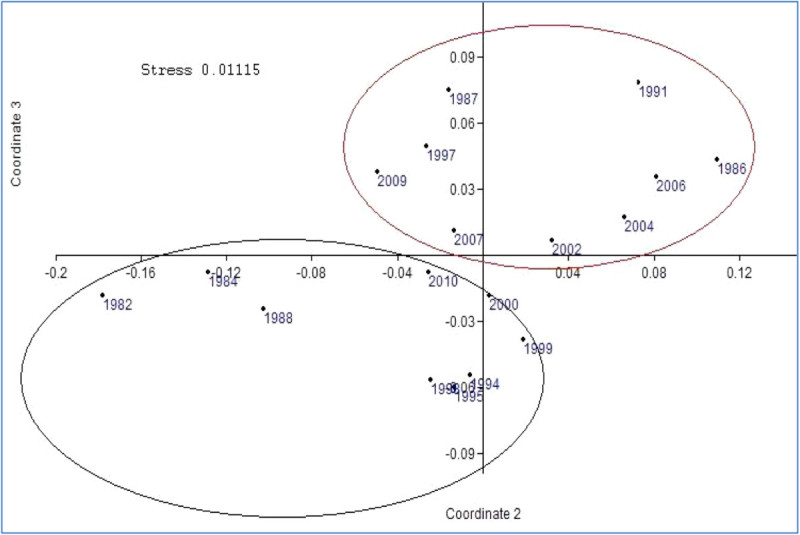
**Non-multidimensional scale plot analysis derived annual observations of Oceanic variables and tuna landings of Indian Ocean.**

Influence of oceanic parameters on the distribution and tuna landings in the Indian Ocean, evaluated by using MDS which described each coordinate, as well as to visualize similarities or differences between El Nino and La Nina years (Figure 11). The similarities among the ENSO event were set up according to coordinate 1 and coordinate 2. The figure illustrates that coordinate A included 1982, 1984, 1988, 1994, 1995, 1998, 2010 and 1999, 2000 (most of the ENSO event from La Nina years) characterize the negative and positive values in multidimensional plot. Coordinate B included 1986, 1987, 1991, 1997, 2002, 2004, 2006 and 2009 (most of the ENSO event from El Nino years) characterize the positive values in multidimensional plot. These results confirmed by the principal components of the similarities performed.

The distribution of tuna in the Indian Ocean and its relation to oceanic structure has been widely discussed (Chen et al. 2005; Song et al. 2009). Our results provide confirmation that the temporal fluctuations of the catches of tuna species in the Indian Ocean during El Nino years, as reported earlier in the Panama bight (United States) Pacific coast (Pedraza and Dıaz-Ochoa 2006), and in the western Pacific (Lehodey et al. 2008). ENSO events seem to be the primary source of variability of tuna landings in the Indian Ocean during 1980–2010. If we accept the first principal components as a good index of environmental effects, about 75.5% of landing variance is accounted by for by simple linear models. Thus, landings in Indian Ocean tend to be optimum SST 25 to 26°C in ENSO event. This evidence of ENSO related zonal displacement of the tuna distribution could allow the prediction of favorable fishing grounds in advance, according to the results of the cross correlation (Lehodey et al. 1997). Climate variability drives seasonal changes in the placement of the most productive fishing grounds (Miller 2007).

## Conclusions

El Nino Southern Oscillation affects ecological patterns and processes in both marine and terrestrial ecosystems. It is observed from the present study that the maximum tuna landings recorded during the weak El Nino years (1425640 t in 2004 and 1456054 t in 2006). It is also observed that SST, Sea level pressure, and Wind actions (U, V and W), are the three environmental variables which are affecting the distribution and abundance of tuna in the Indian Ocean. Surface ocean temperature and food availability are the main factors modified by ENSO impacting on tuna population dynamics (Lehodey et al. 1997). Sea surface temperature explained the highest deviance in generalized additive models and was therefore considered the best habitat predictor in the Indian Ocean followed by SLP and Winds (U, V, W). This relationship can be used to predict (several months in advance of Tuna Forecasting) the region of highest tuna abundance, within a fishing ground extending over the Indian Ocean. A better understanding of the relationships between oceanic environments, distribution and fishing conditions could make utilization of tuna resources more efficient, profitable and sustainable. However, further investigation is required to ascertain the role ENSO in the distribution of tunas in the Indian Ocean. Future research should focus more on longer time series data and investigate the spatial variability of key environmental variables SST and food distribution in the Indian Ocean in relation to tuna distribution and its relationship with climate on different time scales could further improve tuna habitat models. The information obtained should improve capacity to develop fisheries management policies that are resilient and can be adapted to climate change.

## Authors’ original submitted files for images

Below are the links to the authors’ original submitted files for images. Authors’ original file for figure 1 Authors’ original file for figure 2 Authors’ original file for figure 3 Authors’ original file for figure 4 Authors’ original file for figure 5 Authors’ original file for figure 6 Authors’ original file for figure 7 Authors’ original file for figure 8 Authors’ original file for figure 9 Authors’ original file for figure 10 Authors’ original file for figure 11
