# Erratum to: El Nino Southern Oscillation (ENSO) impact on tuna fisheries in Indian Ocean

**DOI:** 10.1186/2193-1801-3-730

**Published:** 2014-12-12

**Authors:** Palanisamy Satheesh Kumar, N Gopalakrishna Pillai, Ushadevi Manjusha

**Affiliations:** Central Marine Fisheries Research Institute, Kochi, Kerala 682 018 India

## Correction

The author list in the original version of this article (Kumar et al. 2014) incorrectly listed the second author’s name as Gopalakrishna N Pillai. The correct form is N Gopalakrishna Pillai.

The publisher would like to apologise for this error.
