# Supplementary figures and images for: El Nino Southern Oscillation (ENSO) impact on tuna fisheries in Indian Ocean
Source: Springerplus. 2014 Oct 9;3(1):591. doi: 10.1186/2193-1801-3-591 (PMC4447736; doi:10.1186/2193-1801-3-591)

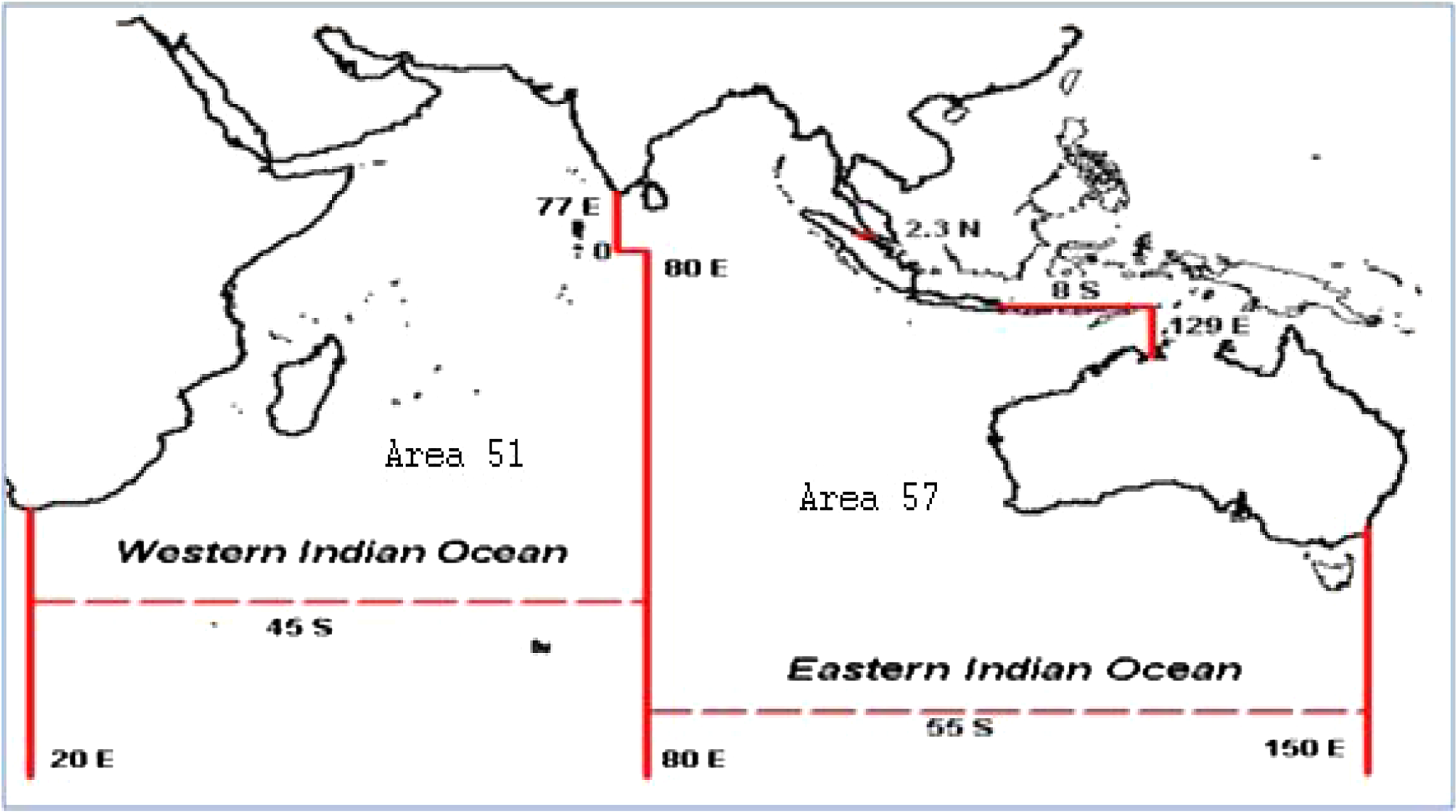

Supplement: Supplementary file 1 — Authors’ original file for figure 1 [file 40064_2014_1562_MOESM1_ESM.tif]

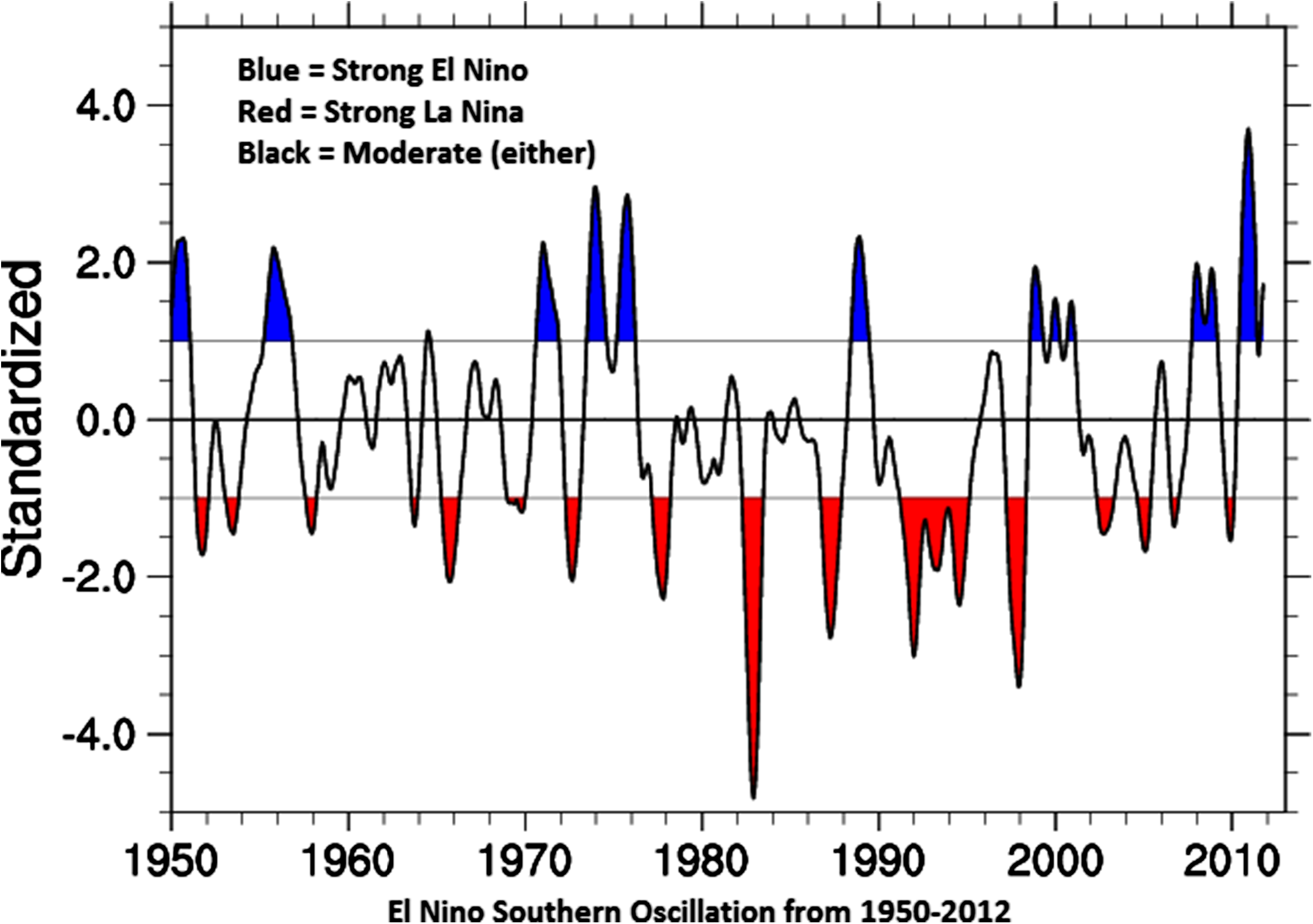

Supplement: Supplementary file 2 — Authors’ original file for figure 2 [file 40064_2014_1562_MOESM2_ESM.tiff]

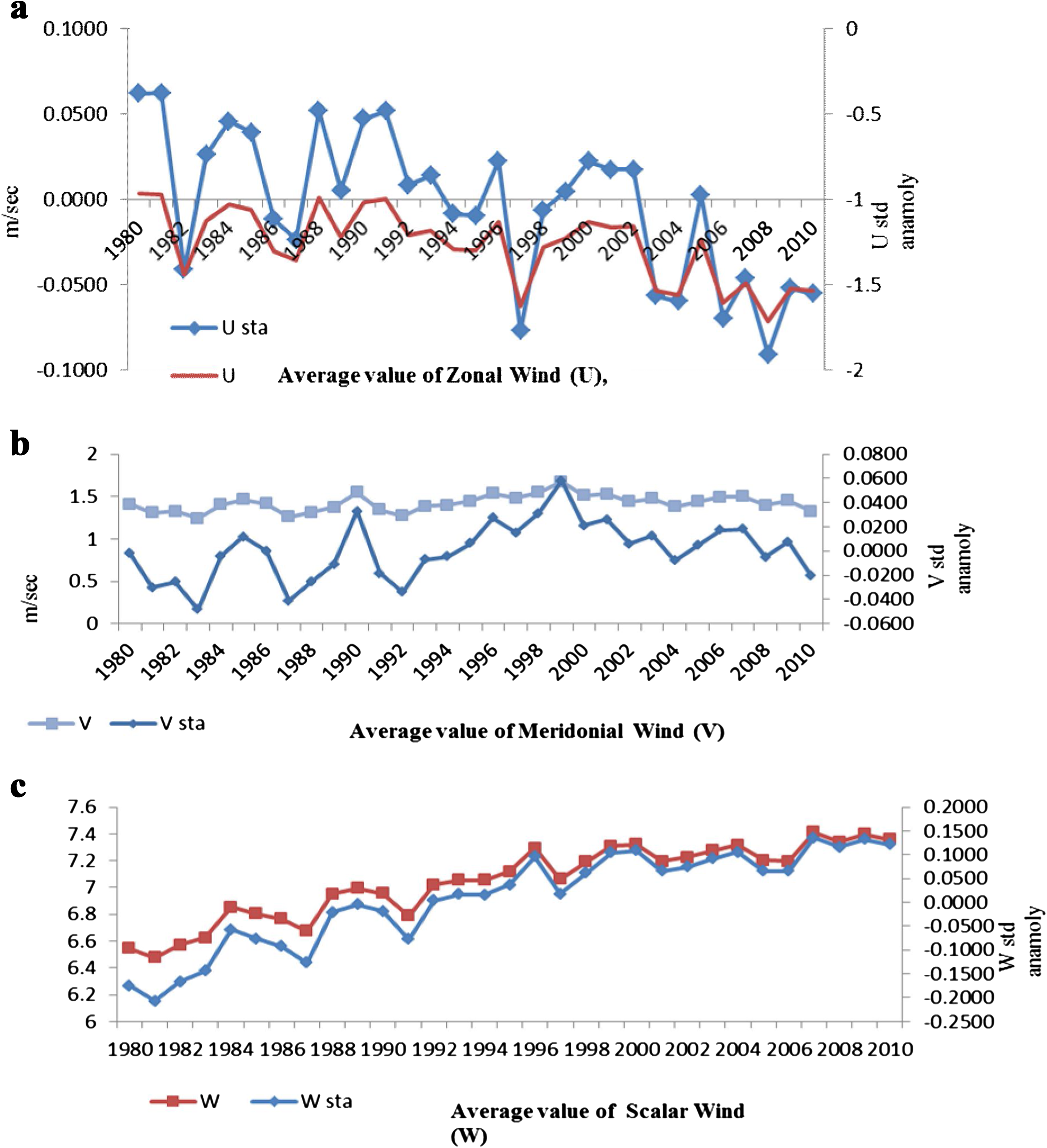

Supplement: Supplementary file 3 — Authors’ original file for figure 3 [file 40064_2014_1562_MOESM3_ESM.tif]

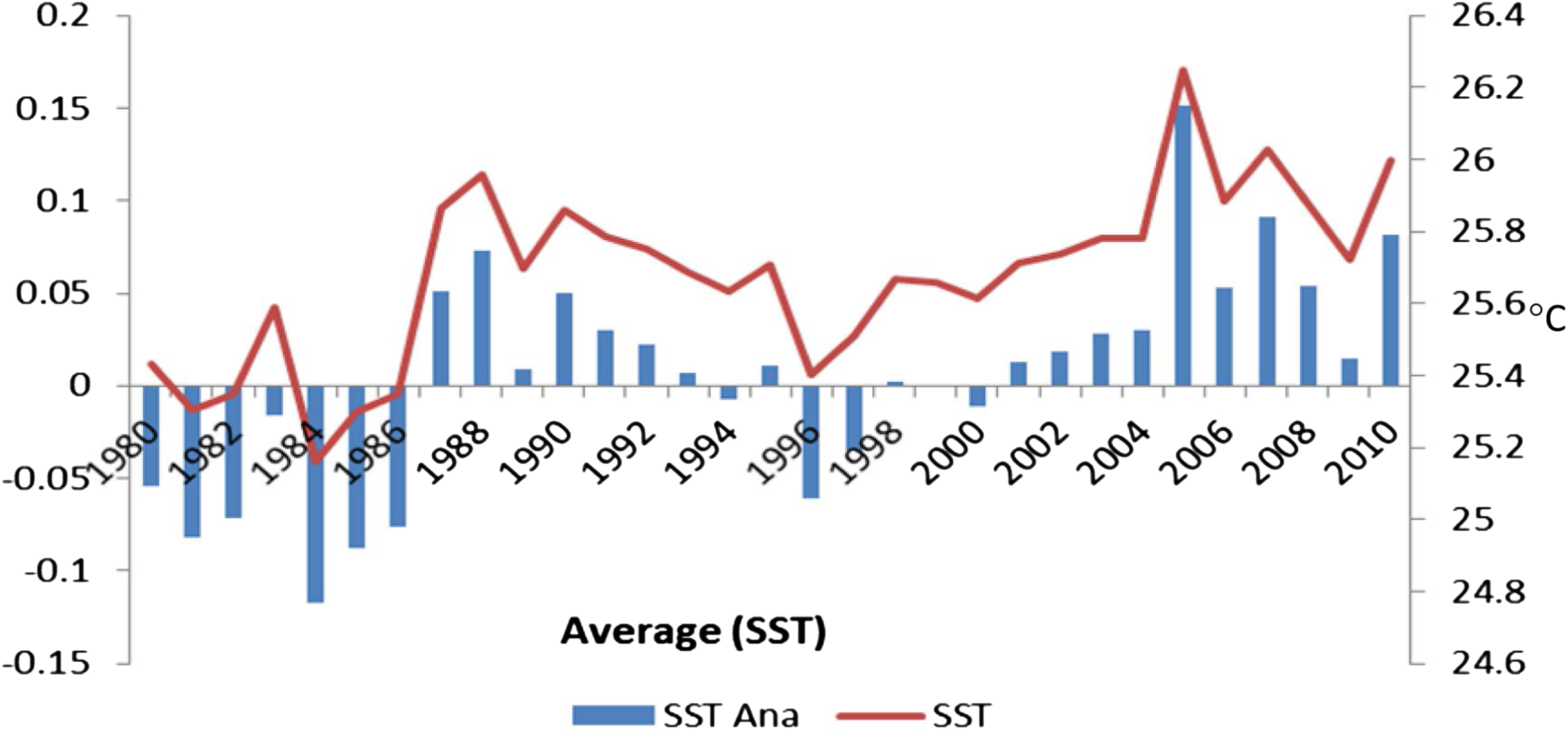

Supplement: Supplementary file 4 — Authors’ original file for figure 4 [file 40064_2014_1562_MOESM4_ESM.tif]

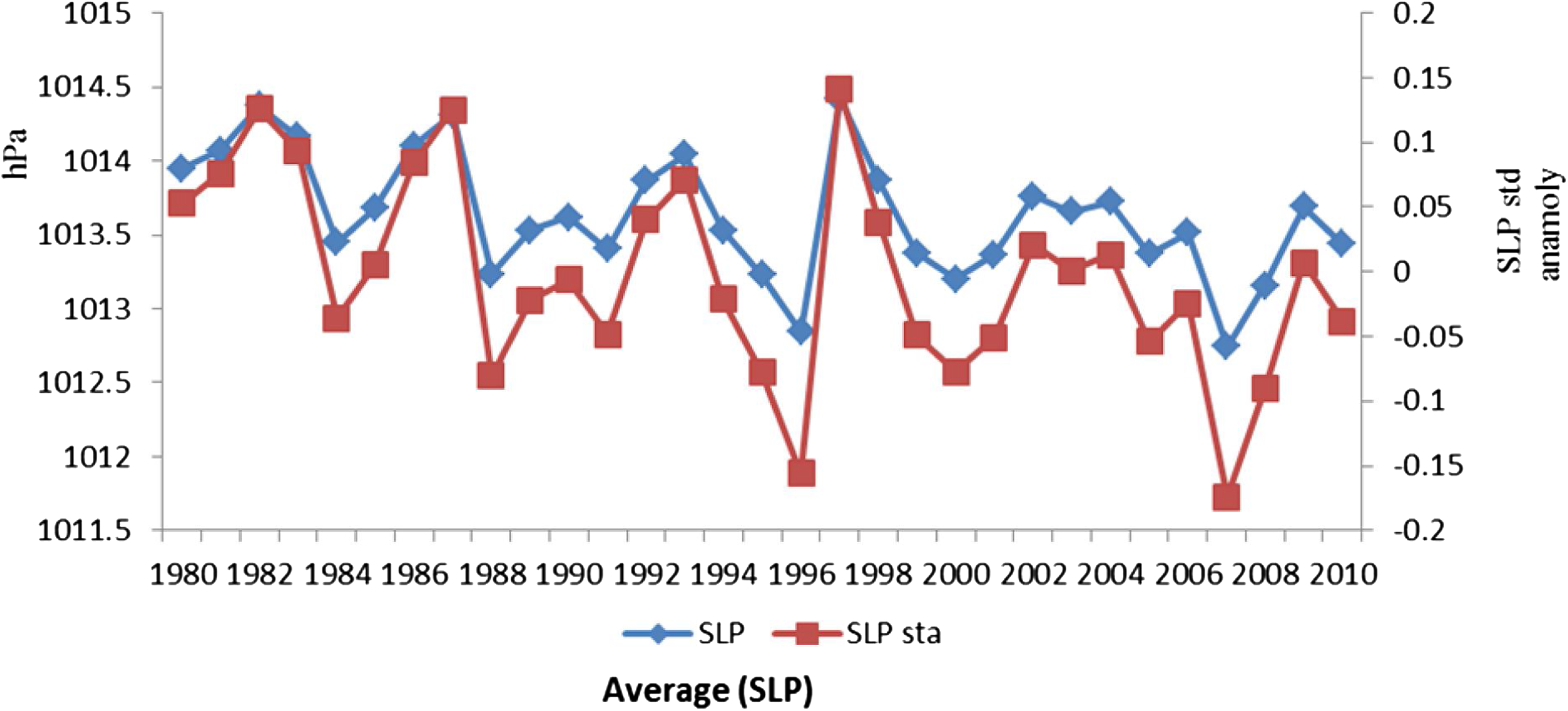

Supplement: Supplementary file 5 — Authors’ original file for figure 5 [file 40064_2014_1562_MOESM5_ESM.tif]

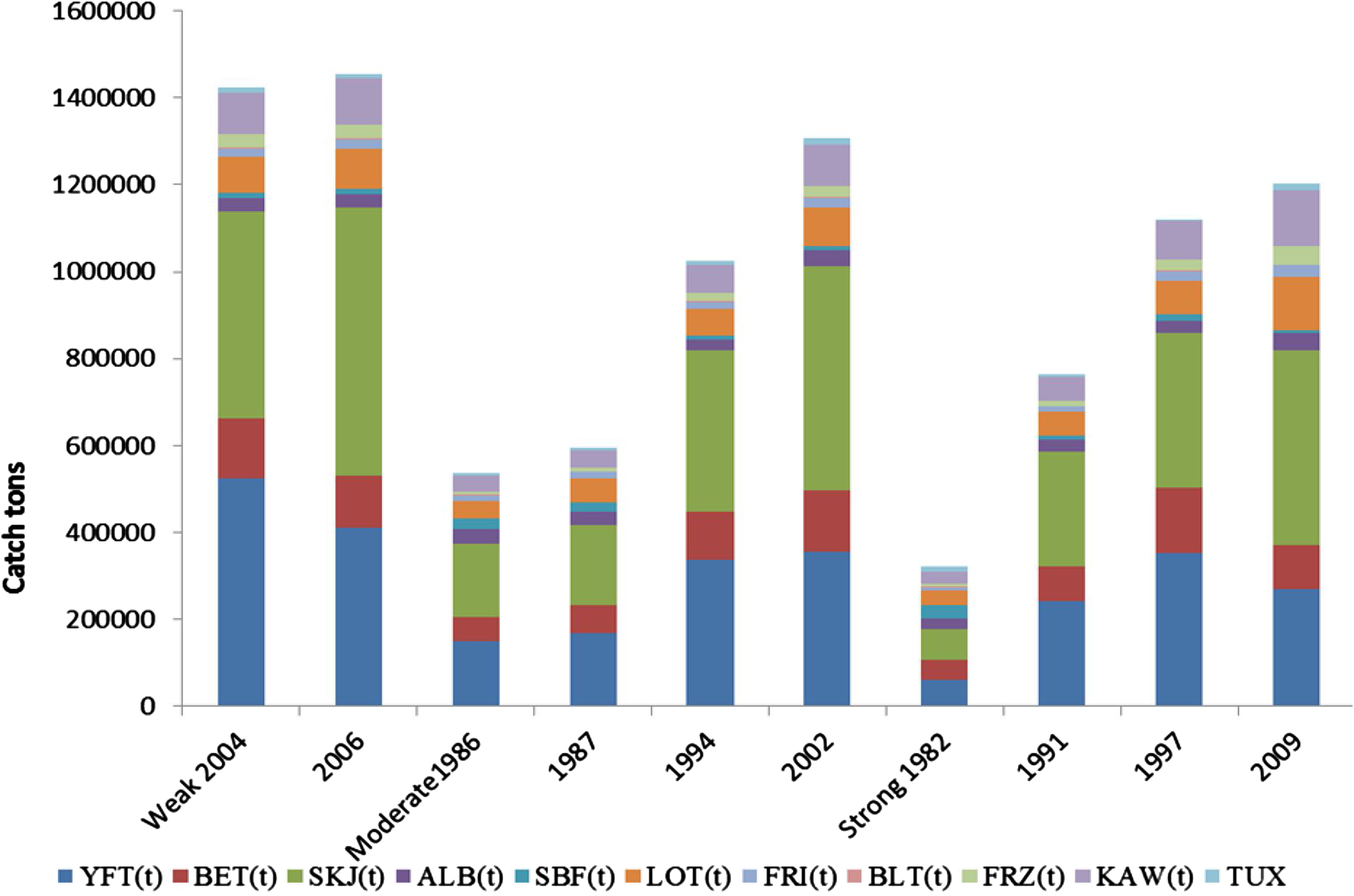

Supplement: Supplementary file 6 — Authors’ original file for figure 6 [file 40064_2014_1562_MOESM6_ESM.tif]

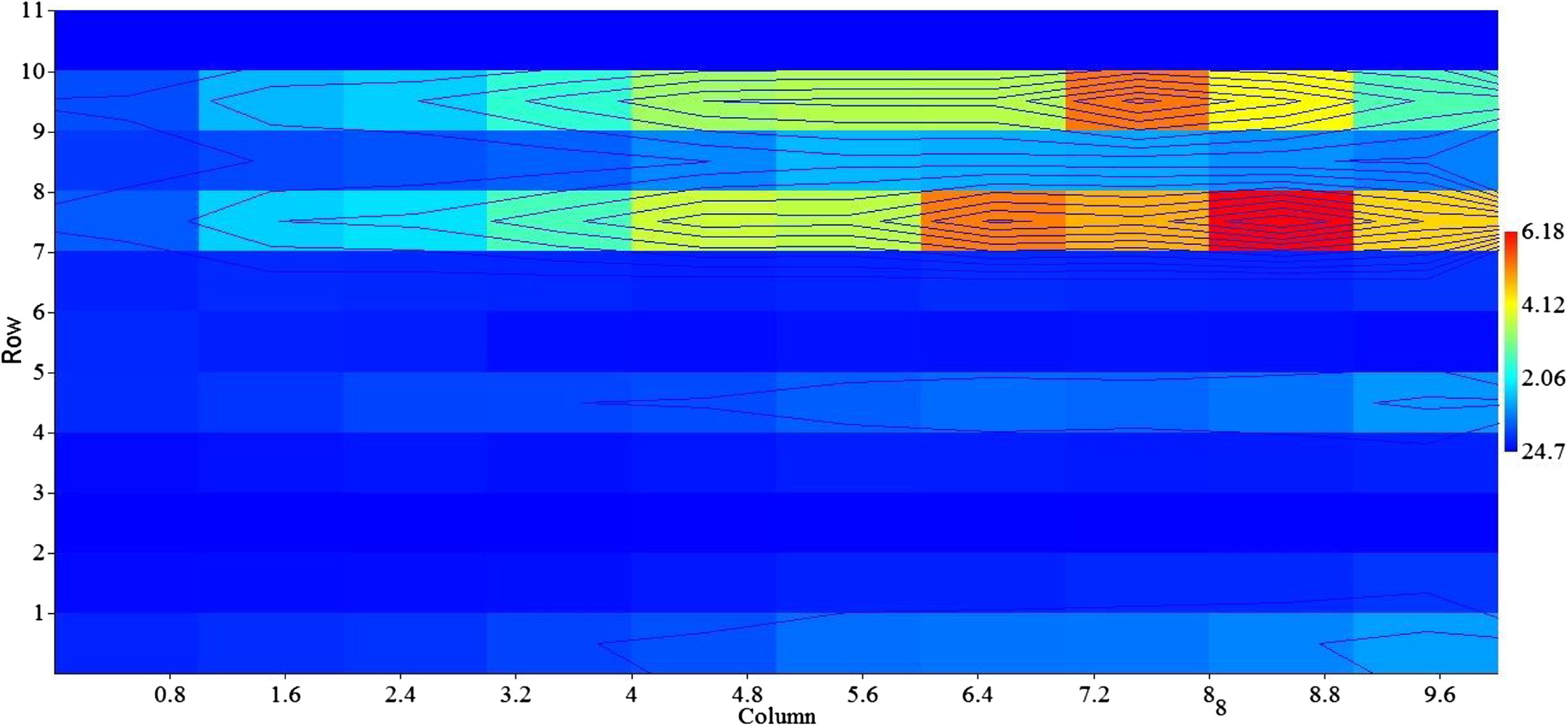

Supplement: Supplementary file 7 — Authors’ original file for figure 7 [file 40064_2014_1562_MOESM7_ESM.tif]

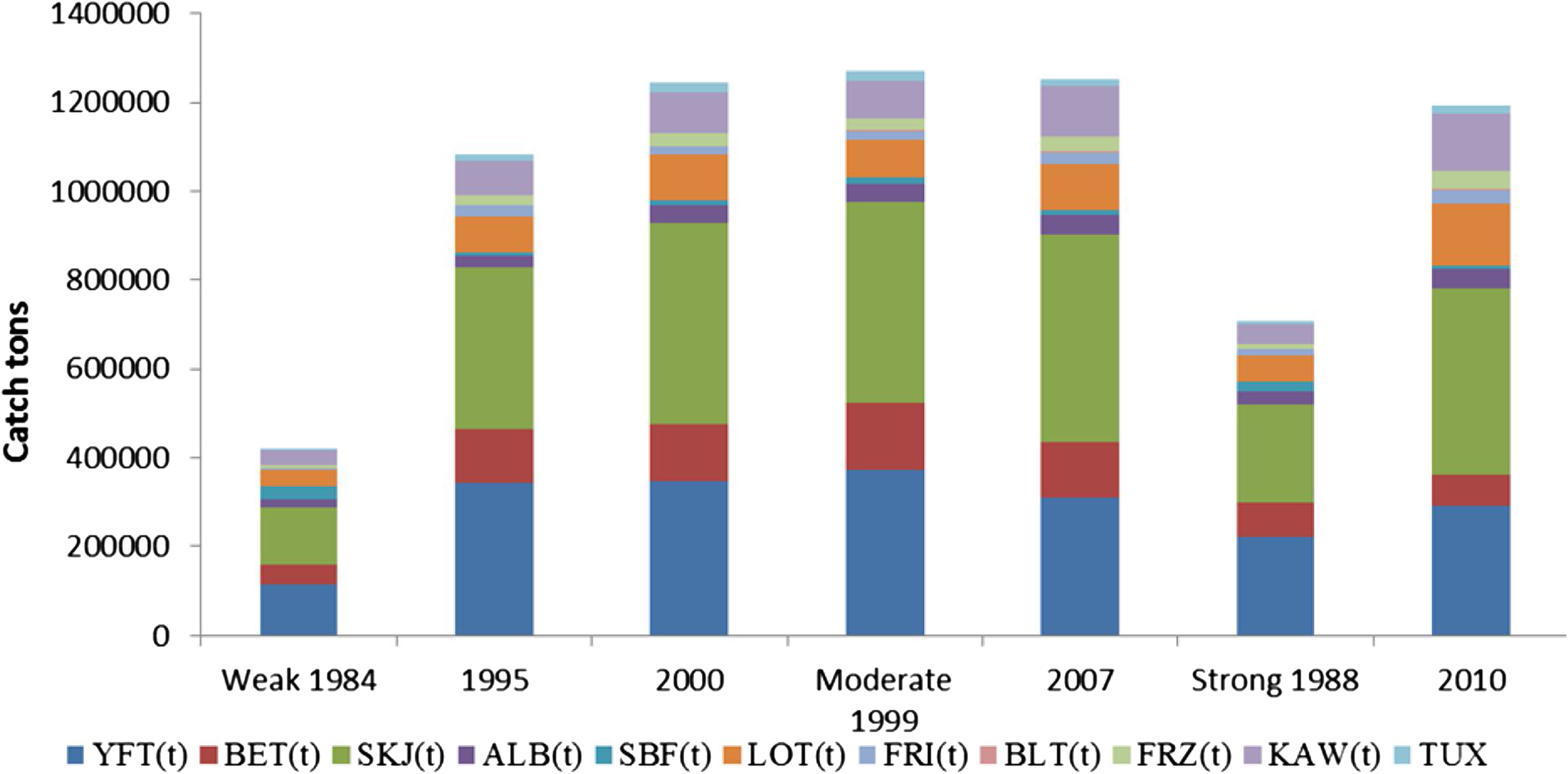

Supplement: Supplementary file 8 — Authors’ original file for figure 8 [file 40064_2014_1562_MOESM8_ESM.tif]

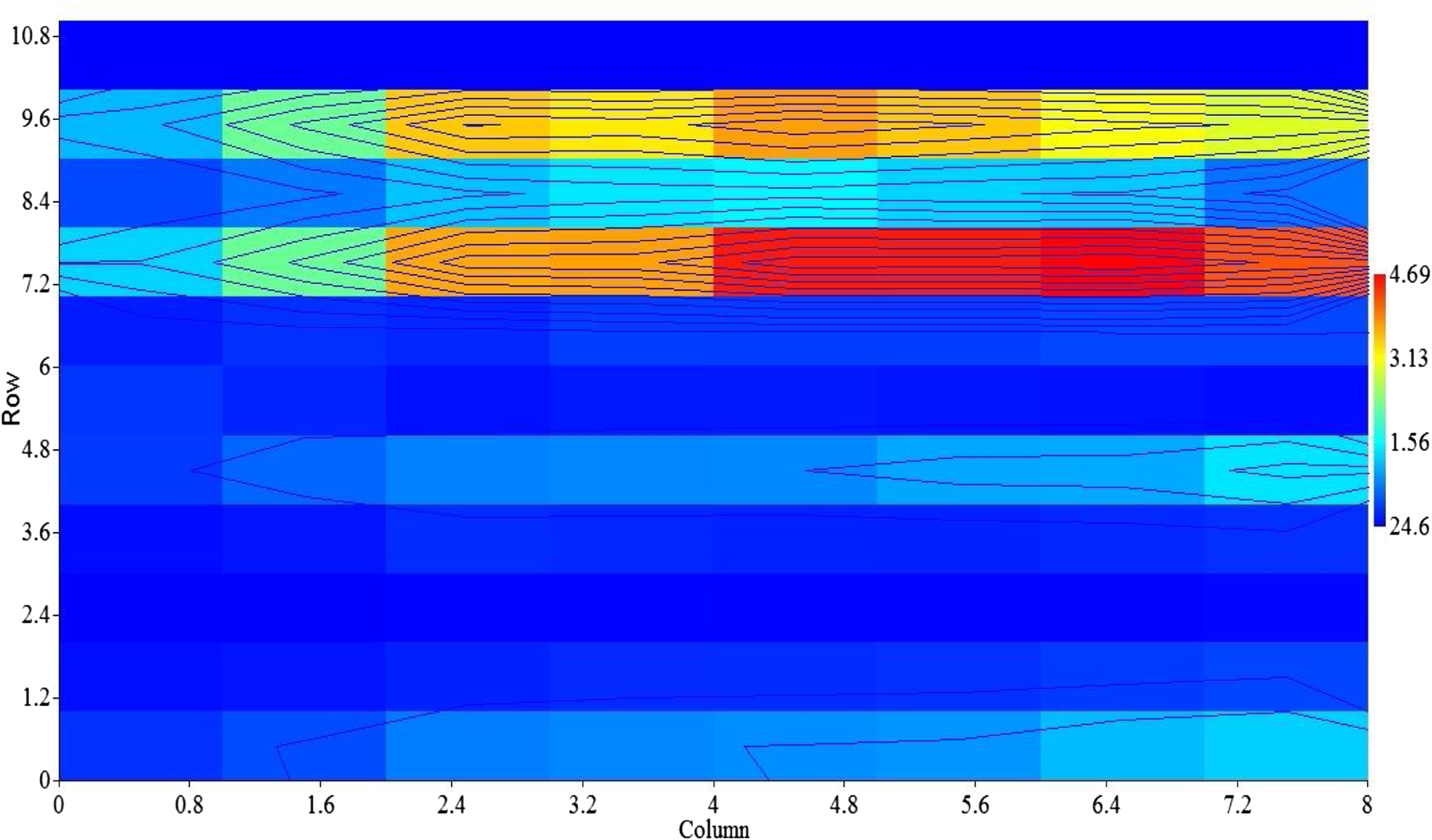

Supplement: Supplementary file 9 — Authors’ original file for figure 9 [file 40064_2014_1562_MOESM9_ESM.tif]

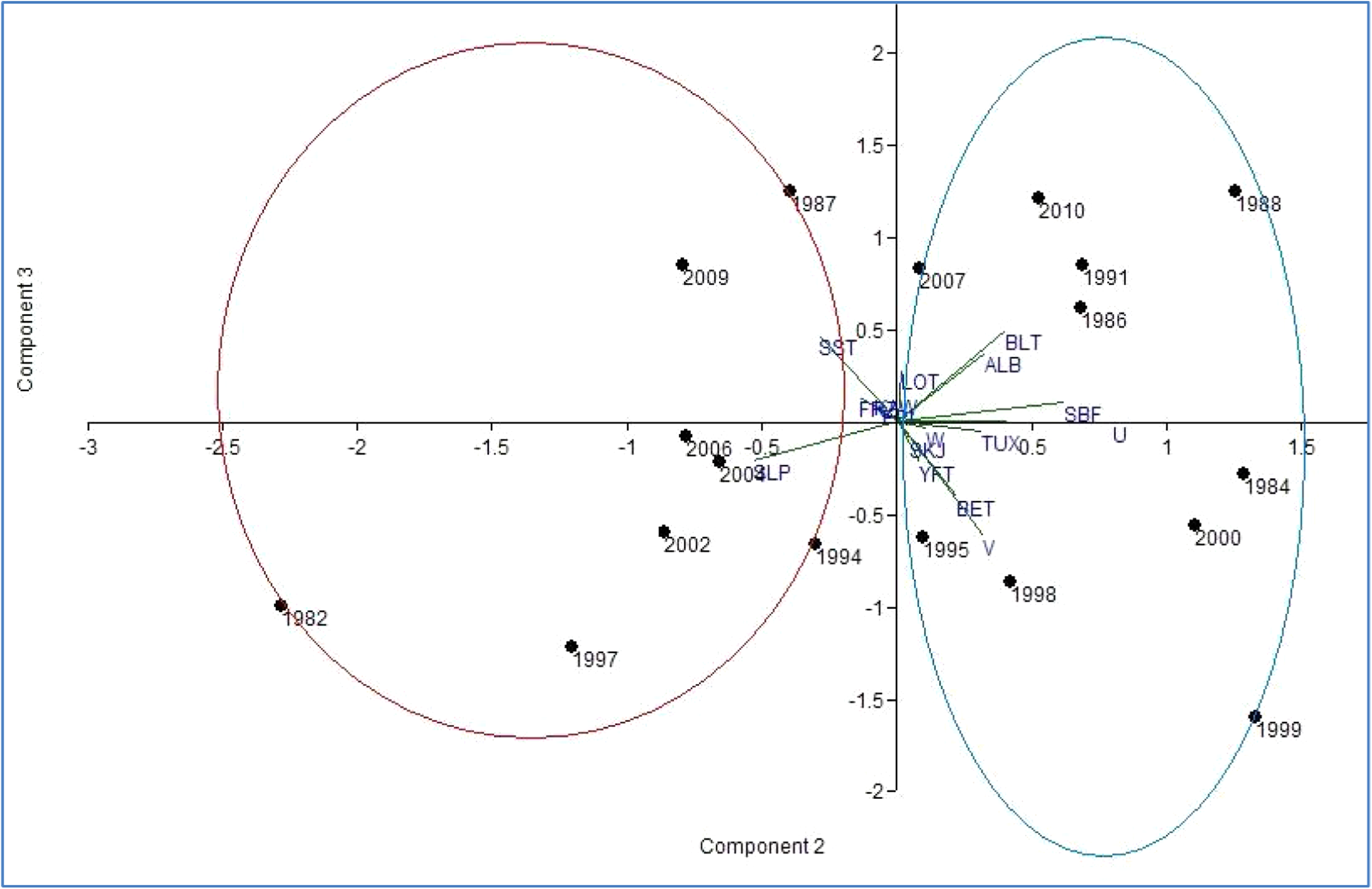

Supplement: Supplementary file 10 — Authors’ original file for figure 10 [file 40064_2014_1562_MOESM10_ESM.tif]

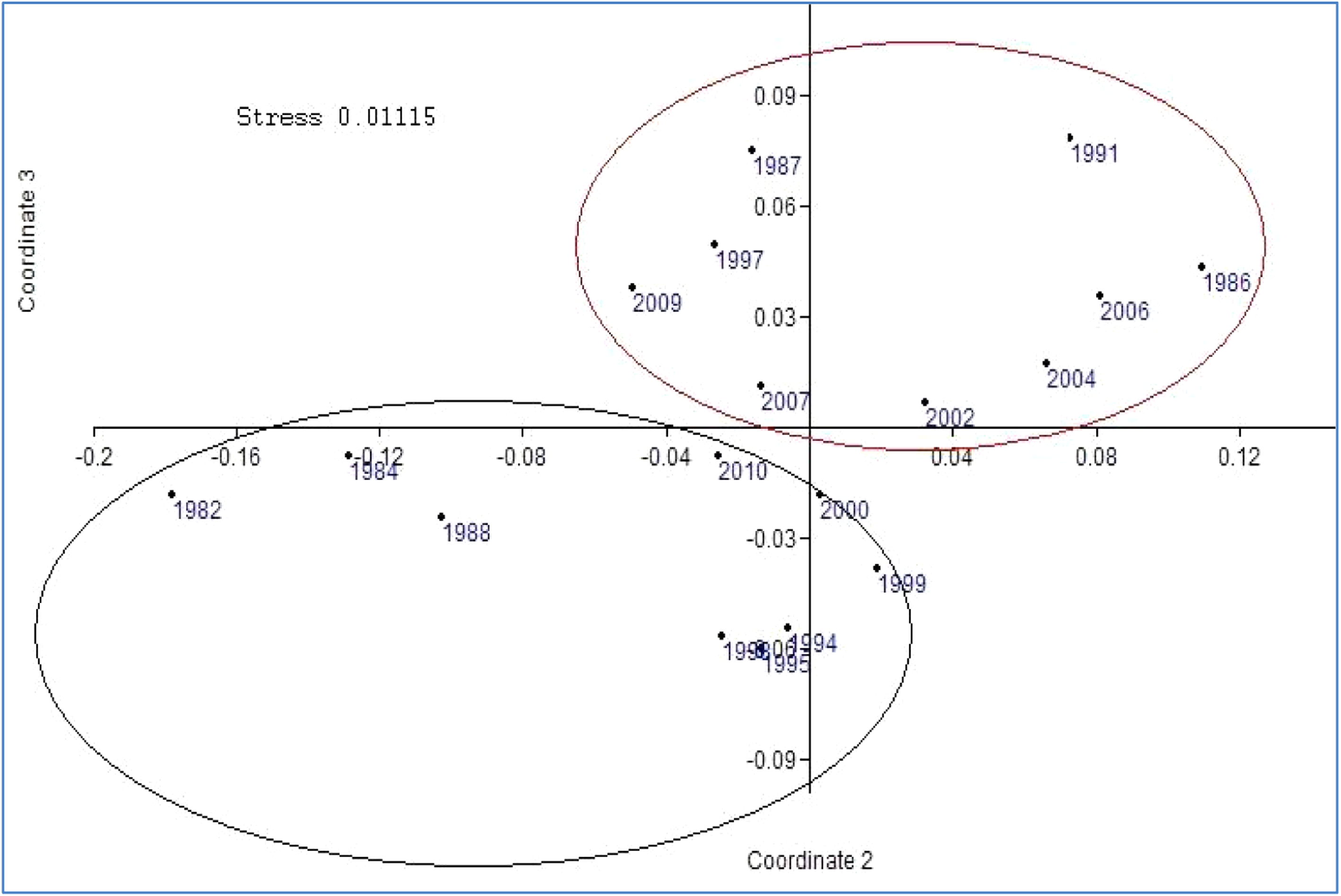

Supplement: Supplementary file 11 — Authors’ original file for figure 11 [file 40064_2014_1562_MOESM11_ESM.tif]
